# Supplementary material for: Solid Fe Resources Separated from Rolling Oil Sludge for CO Oxidation
Source: Int J Mol Sci. 2022 Oct 12;23(20):12134. doi: 10.3390/ijms232012134 (PMC9602949; doi:10.3390/ijms232012134)
Supplement: Supplementary file 1 [file ijms-23-12134-s001.zip › ijms-1944143-supplementary.pdf]

Supporting information for

Solid Fe Resources Separated from Rolling Oil Sludge for CO Oxidation

Wei Gao †, *Sai Tang* †, *Ting Wu*, *Jianhong Wu*, *Kai Cheng* * *and Minggui Xia* *

(Hubei Key Laboratory of Biomass Fibers and Eco-dyeing & Finishing, School of Chemistry and Chemical Engineering, Wuhan Textile University, Wuhan 430200, China)

* Corresponding authors: Kai Cheng, E-mail: kcheng55@126.com;

Minggui Xia, E-mail: mingguixia1@163.com.

† The two authors have equal contribution as the first author

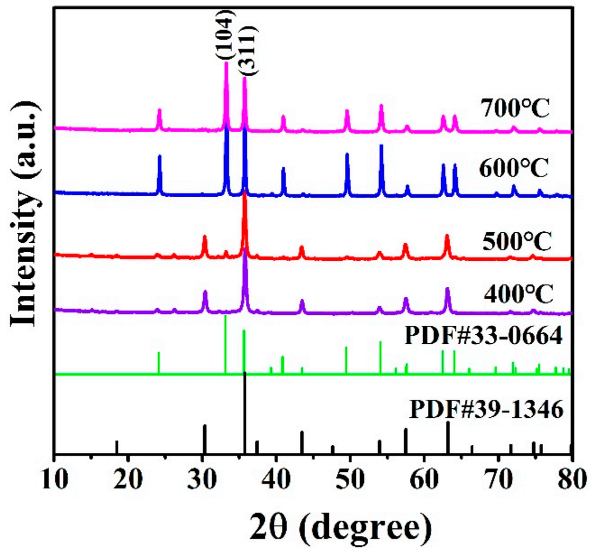

Figure S1. XRD patterns of Fe_2O_3-H under different calcination temperatures

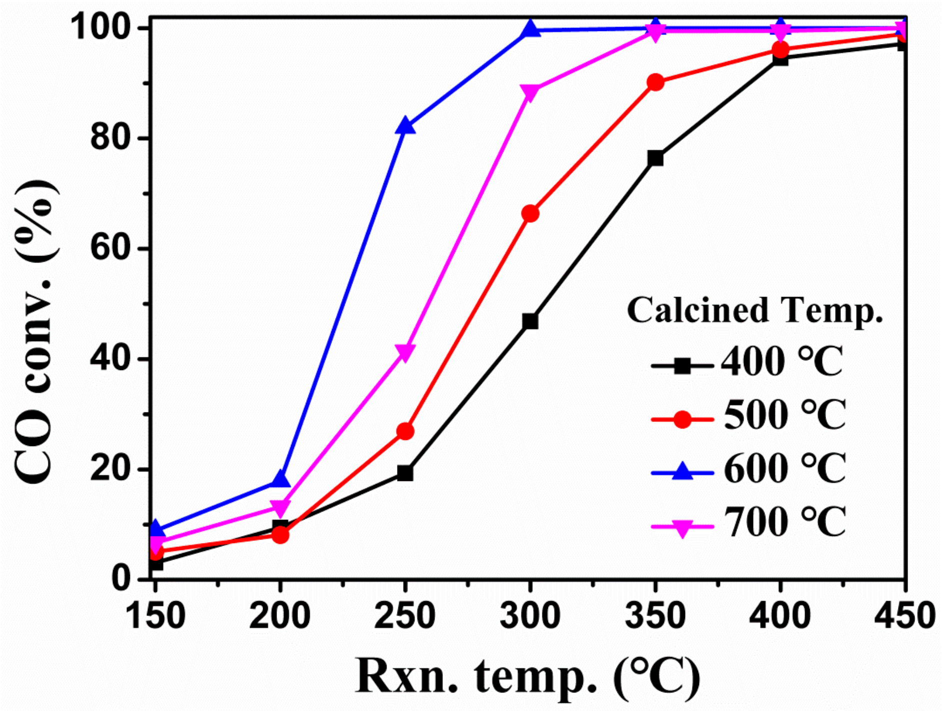

Figure S2. Effect of different calcination temperatures on CO oxidation activity over Fe_2O_3-H sample

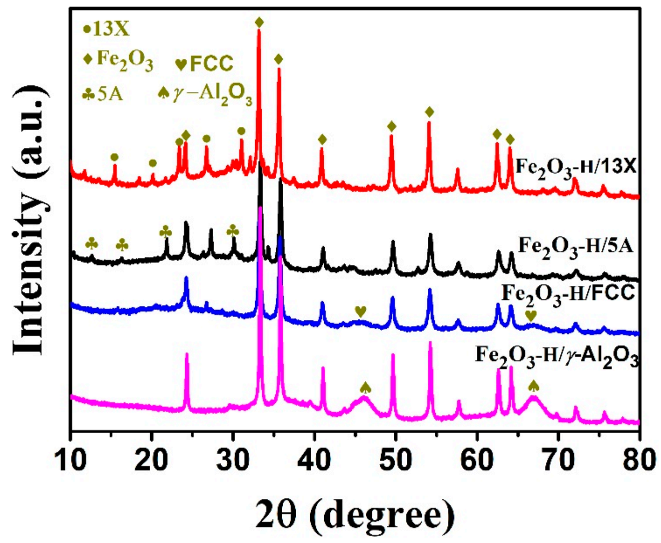

Figure S3. XRD patterns of Fe_2O_3-H supported on 13X, 5A, FCC and γ-Al_2O_3 carriers.

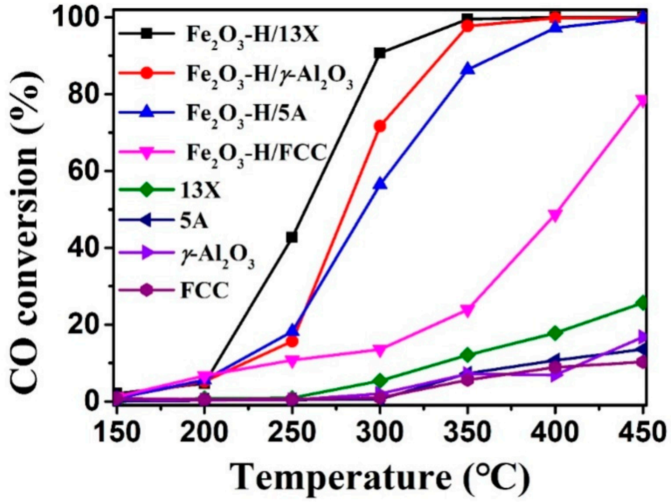

Figure S4. Effect of different supports on CO oxidation activity over supported-Fe_2O_3-H catalysts

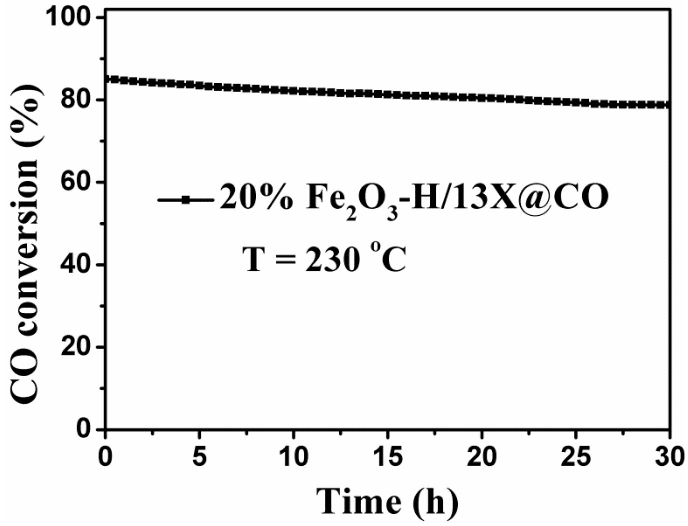

Figure S5. Evolution of CO conversion on the 20% Fe_2O_3-H/13X@CO at 230 ºC during 30 h
